# Affective and Enjoyment Responses to Sprint Interval Exercise at Different Hypoxia Levels

**DOI:** 10.3390/ijerph18158171

**Published:** 2021-08-02

**Authors:** Zhaowei Kong, Mingzhu Hu, Shengyan Sun, Liye Zou, Qingde Shi, Yubo Jiao, Jinlei Nie

**Affiliations:** 1 Faculty of Education, University of Macau, Macao 999078, China; zwkong@um.edu.mo (Z.K.); mb84856@um.edu.mo (Y.J.); 2 Institute of Physical Education, Huzhou University, Huzhou 313000, China; antun0605@163.com; 3 Exercise Psychophysiology Laboratory, Institute of KEEP Collaborative Innovation, School of Psychology, Shenzhen University, Shenzhen 518060, China; liyezou123@gmail.com; 4 School of Health Sciences and Sports, Macao Polytechnic Institute, Macao 999078, China; qdshi@ipm.edu.mo (Q.S.); jnie@ipm.edu.mo (J.N.)

**Keywords:** interval exercise, high-intensity interval training, pleasure, exercise adherence

## Abstract

Benefits of performing sprint interval training (SIT) under hypoxic conditions on improving cardiorespiratory fitness and body composition have been well-documented, yet data is still lacking regarding affective responses to SIT under hypoxia. This study aimed to compare affective responses to SIT exercise under different oxygen conditions. Nineteen active males participated in three sessions of acute SIT exercise (20 repetitions of 6 s of all-out cycling bouts interspersed with 15 s of passive recovery) under conditions of normobaric normoxia (SL: PIO_2_ 150 mmHg, FIO_2_ 0.209), moderate hypoxia (MH: PIO_2_ 117 mmHg, FIO_2_ 0.154, simulating an altitude corresponding to 2500 m), and severe hypoxia (SH: PIO_2_ 87 mmHg, FIO_2_ 0.112, simulating an altitude of 5000 m) in a randomized order. Perceived exertions (RPE), affect, activation, and enjoyment responses were recorded before and immediately after each SIT session. There were no significant differences across the three conditions in RPE or the measurements of affective responses, despite a statistically lower SpO_2_ (%) in severe hypoxia. Participants maintained a positive affect valence and reported increased activation in all the three SIT conditions. Additionally, participants experienced a medium level of enjoyment after exercise as indicated by the exercise enjoyment scale (EES) and physical activity enjoyment scale (PACES). These results indicated that performing short duration SIT exercise under severe hypoxia could be perceived as pleasurable and enjoyable as performing it under normoxia in active male population.

## 1. Introduction

Training in hypoxia has been widely adopted as an effective strategy to enhance exercise performance and physical fitness in athletes for decades [1]. More recently, this training strategy has shown its beneficial effects on cardio-metabolic health and weight management in untrained individuals [2]. Compared to training at sea level, hypoxic training could induce additive improvements in health and fitness, as reported in previous studies [3,4,5]. However, health improvements provided by hypoxic training seem to be influenced by training intensity. A meta-analysis [6] suggested that high-intensity interval training (HIIT), especially sprint interval training (SIT) with supramaximal intensity, appeared to be a better combination with hypoxia to elicit meaningful performance improvements compared to low-intensity training. Our previous study which focused on overweight individuals also revealed a significantly additive effect of SIT (i.e., 60 repetitions of 8 s sprinting interspersed with 12 s recovery) on cardiorespiratory fitness under normobaric hypoxia (FIO_2_: 0.15) compared to identical SIT under normoxia (FIO_2_: 0.21) [5]. Therefore, combining SIT with hypoxia could be worth consideration for individuals who want to improve health and fitness in an efficient manner. 

Although the efficacy of SIT under hypoxia in improving exercise capacity and health status has been well-documented in the existing literature, affective responses to this type of training strategy have not been examined. Affective responses (i.e., enjoyment, affect) are exercise-induced feelings that potentially associate with exercise automaticity (i.e., willingness to exercise) and future exercise adherence [7,8,9]. Despite that adverse affective responses could more likely appear in performing exercise with supramaximal intensity according to the dual-mode theory [10], recent reviews suggested that, in most cases, enjoyment responses to HIIT were similar compared to moderate intensity continuous trainings when performed in normoxia [11,12,13]. It seems that the strategy of alternating work and recovery intervals and relatively short work durations (i.e., <10 s) could mitigate the unpleasant feelings caused by maximal or supramaximal intensity in SIT [14]. Nevertheless, performing “all-out” interval training under hypoxia could result in greater physiological stress and perceived discomfort compared to training in normoxia [15], and thus may negatively impact affective responses. Additionally, although training under more severe hypoxia (e.g., altitude at 5000 m) might lead to greater physiological adaptions [16], exacerbated exercise-related sensations could also be triggered. Extreme reduction of oxygen availability inevitably limits cardiorespiratory capacity, increases the likelihood to premature fatigue, and elevates blood lactate accumulations [17,18,19]. The physiological stress might lead to larger perceptual strains such as higher perceived discomfort and difficulty breathing during sprint exercise under severe hypoxia [15,20]. However, given that no study to date has investigated the affective responses to SIT exercise under different hypoxia, whether affective responses would be influenced by hypoxic conditions remains unknown.

As such, the current study aimed to investigate and compare acute affective responses to a SIT protocol involving sprint effort under normobaric normoxia (PIO_2_ = 150 mmHg, FIO_2_ = 0.209), moderate hypoxia (PIO_2_ = 117 mmHg, FIO_2_ = 0.154), and severe hypoxia (PIO_2_ = 87 mmHg, FIO_2_ = 0.112). Main outcomes of affective responses included enjoyment, affect, and arousal post exercise. Given the previous studies that suggested that performing SIT could induce unpleasant feelings due to its supramaximal intensity and the findings that greater perceptual strains and higher perceived discomfort could be generated during exercise under marked hypoxia conditions, it was hypothesized that SIT exercise under severe hypoxia would elicit more negative affective responses compared to moderate hypoxia and normoxia. 

## 2. Materials and Methods

### 2.1. Participants

The study was approved by the Research Ethics Panel of the University of Macau (approval number: MYRG2015-00223-FED) and all experimental procedures were in accordance with the declaration of Helsinki. Prior to recruitment, a required sample size was estimated (*t*-test, point biserial correlation model) using power analysis (G*Power software, version 3.1). Based on the effect size reported by a meta-analysis of studies that examined physical activity enjoyment scale (PACES) responses to different types of SIT [12], a medium effect size of 0.5, an alpha criterion of 0.05, and an assumed power (1 − β) of 0.8 were used for power analysis, and the result suggested that a total of 20 participants were required in this study.

The exclusion criteria for participation included residence at altitudes above 1000 m or exposure to an altitude above 1000 m in past six months before the experiment; having prior experience in hypoxic training; smokers; taking medication or having any physical barriers to performing SIT exercise under hypoxia. Importantly, given that performing ‘all-out’ SIT could be very demanding under severe hypoxia, recreational active men were included with maximal oxygen uptake ($\overset{˙}{V}$O_2max_) ranging from 40 to 50 ml∙kg^−1^∙min^−1^ after an incremental ramp test which is similar to our previous study to decide $\overset{˙}{V}$O_2max_ [21]. Briefly, subsequent to a general warm-up, each individual performed a graded exercise-cycling test on a cycle ergometer at an initial workload of 60 Watts (W). The exercise workload was increased by 30 W for every 2 min (with a cycling cadence of 60 ± 5 rpm) until participants’ volitional exhaustion. Data concerning ventilation were recorded throughout the test. The $\overset{˙}{V}$O_2max_ was determined as the average of the highest 30 s value of $\overset{˙}{V}$O_2_ in the last stage. 

Twenty-two participants were recruited to participate in this study. Three individuals dropped out because of personal reasons. Finally, 19 recreational active males (age 20.7 ± 1.8 y, height 178.4 ± 8.1 cm, weight 69.8 ± 11.0 kg, $\overset{˙}{V}$O_2max_ 42.7 ± 1.4 ml∙kg^−1^∙min^−1^) completed all the required testing procedures.

### 2.2. Experimental Design

This within-subjects design study included a preliminary stage and three main experimental trials. During the preliminary stage, eligible participants were familiarized with the experimental procedures and practiced the SIT protocol. After the preliminary stage, the participants accomplished three experimental trials under different oxygen concentrations, namely, a SIT trial under normoxia at sea level (SL, PIO_2_ = 150 mmHg, FIO_2_ = 0.209), a SIT trial under moderate hypoxia (MH, PIO_2_ =117 mmHg, FIO_2_ = 0.154, simulating an altitude corresponding to 2500 m) and a SIT trial under severe hypoxia (SH, PIO_2_ = 87 mmHg, FIO_2_ = 0.112, simulated at an altitude of 5000 m). 

The three SIT trials were carried out at the same time of the day (6:00–8:10 p.m.) on three separate weeks at the lab, in which the room temperature (22 ℃) and humidity (50–60%) were well controlled. Two experienced research assistants supervised the experimental process and recorded the exercise data. In order to exclude the potential influence of diet and daily physical activity on outcome variables, participants were instructed to refrain from coffee and alcohol, as well as strenuous exercise 48 h before the days of experiment. Routine physical activities were examined on one day before and on the day of experiment using validated pedometers (Yamax Digi-Walker SW-200, Tokyo, Japan).

On the day of trials, two standard meals (lunch and dinner) were provided to each participant with ~680 kcal, in which carbohydrate, protein and fat accounted for approximately 60%, 10%, and 30% of total energy intake. Participants finished their lunch around 1:00 p.m., while they were required to arrive at the lab before 6:00 p.m. and thereafter completed the provided dinner. At 7:30 p.m., participants were fitted with a facemask connected to a modified gas mixing system (Everest Summit II Hypoxic Generator, New York, NY, USA) and they rested quietly in a seated position for 30 min. The normoxic or hypoxic gas mixtures were generated by this system and were delivered to participants through tubes and breathing mask. At 8:00 p.m., participants were asked to fill the Feeling Scale (FS), Felt Arousal Scale (FAS), and Exercise Enjoyment Scale (EES) as baseline, and then they performed SIT exercise under either one of the three oxygen conditions. The three trials were assigned in a randomized and counterbalanced order and were interspersed by seven days as wash-out periods. Participants were blinded to the normoxic or hypoxic condition of the experimental trials. Scores of the FS, FAS, and EES were collected again immediately post the exercise trial. The PACES was conducted after the complement of EES.

### 2.3. Main Outcome Measures

Affective valence was measured through FS pre and immediately post the exercise trial. The FS is an 11-point, single item scale ranging from −5 (very bad) to +5 (very good) to indicate the feelings of pleasure/displeasure. Perceived activation before and after the exercise was indicated by scores of FAS, which is a 6-point single item scale demonstrating low activation (1 point) to high activation (6 points). The FS and the FAS have been shown to be valid and reliable instruments and have been widely used in the research field to measure exercise-induced affective responses [22]. Affect valences indicated by FS demonstrate global and core affective responses to exercise through initial determinations of good or bad, while specific domains of affective responses might not be captured through FS. Nevertheless, the combination of FAS with FS to generate circumplex model of affect could provide more information on specific affective state (e.g., being pleasant and having high activation indicating a feeling of excitement) [23]. EES [24] and PACES [25] were applied to measure exercise enjoyment. EES has been used to measure changes of enjoyment during acute exercise bout because of its simplicity, while PACES taken only post-exercise requires recall of the exercise experience and demonstrates memory of enjoyment during previous exercise [23]. The EES is a single item, 7-point scale to inquire “how much fun you are having regarding the exercise session”, which indicates an overall feeling of enjoying or not enjoying. The PACES questionnaire is a 7-point 18-item bipolar scale (11 items are scored reversely) in which respondents recall the feelings and enjoyment level towards the exercise they have done. The total scores of PACES are between 18 and 126, with higher scores indicating greater enjoyment. 

### 2.4. Sprint Interval Exercise 

The SIT protocol included 5-min warm up at 60 W with a cycling cadence of 60 rpm, a 7-min SIT trial and a 3-min cool down at a rate of 60 rpm without any workload. The 7-min SIT trial consisted of 20 repetitions of 6 s of all-out cycling bouts interspersed with 15 s of passive recovery. It has been reported that when the work duration was fixed at 6 s, SIT protocols with 15 s recovery could impose higher metabolic stress on the oxidative system compared to SIT protocols with longer recovery durations (i.e., 30 s and 60 s) [26]. More importantly, SIT with fewer sprint repetitions and shorter sprint duration could facilitate the time-efficiency while not reducing improvements in cardiorespiratory fitness according to a recent review [27]. Therefore, a modified SIT protocol with fewer repetitions and shorter sprint durations was incorporated in the current study (i.e., 20 × 6 s all-out sprints interspersed with 15 s rests) compared to that adopted in our previous work (i.e., 60 × 8 s maximal cycling interspersed with 12 s recovery periods) [27].

During the SIT trials, participants pedaled maximally against a load equivalent to 7.5% of body weight during the 6 s work durations, and underwent passive recovery during the 15s rest periods on a cycle ergometer (Monark 839E, Vansbro, Sweden). Heart rate (HR, Polar F4M BLK, Finland), peripheral oxygen saturation (SpO_2_, Radical-7 Pulse CO-Oximeter, Masimo, Irvine, CA, USA), and ratings of perceived exertion (RPE; 0–10 Modified Borg Dyspnea scale) were recorded before and immediately after every five sprint bouts. After the completion of 3-min cool down subsequent to SIT exercise, the participants filled the FS, FAS, EES, PACES scales again. Moreover, they were required to guess the oxygen condition they were assigned to, by asking “Under what condition do you think you were exercising, normoxia or hypoxia (2500 m or 5000 m)?”

### 2.5. Statistical Analysis

The PASW software (Release 22.0, IBM, New York, NY, USA) was used for statistical analyses. Before the main analyses, the Shapiro–Wilk tests were conducted to confirm that the outcome variables were normally distributed. Two-way repeated measures ANOVA analyses were performed to determine the main effects of exercise (pre- and post-SIT) and oxygen conditions (FIO_2_: 0.209, 0.154 and 0.112), and interaction effects (exercise × condition) on psychological (i.e., FS, FAS, and ES) and physiological parameters (i.e., HR, RPE, and SpO_2_). Tukey’s test was used to compare the condition differences when there was significant interaction effect and partial eta squared (*η_p_^2^*) was calculated as the measure of effect size. For *η_p_^2^*, Cohen’s benchmarks of 0.01 (small), 0.06 (medium), and 0.14 (large) were used to evaluate the size of the effect [28]. One-way repeated measures ANOVA analyses were carried out to compare whether there were differences in power outputs during exercise, and PACES after exercise among different conditions. The average of the pre and post standard deviations (SDs) was used as the standardizer to compute the d-value effect sizes [29]. The d-value scores of 0.2, 0.5, and ˃0.8 were classified as small, moderate, and large, respectively [30]. All data were expressed as mean ±SD. Statistics generating *p*-values less than 0.05 were considered as providing some statistical evidence against a null outcome.

## 3. Results

### 3.1. Daily Physical Activity and Physiological Responses

No statistically detectible differences in routine physical activities were found among the three trials one day before (SL: MH: SH = 8813 ± 2945: 8436 ± 4685: 8703 ± 4237 steps) and on the day of experiment (SL: MH: SH = 7392 ± 3759: 6498 ± 3781: 7494 ± 2816 steps).

Eight participants (58%) during SL, 13 (32%) during MH and 11 (42%) during SH provided a correct answer about the condition assignment, suggesting that the participants were generally not able to guess the oxygen concentration of the SIT conditions. 

**Figure 1:**
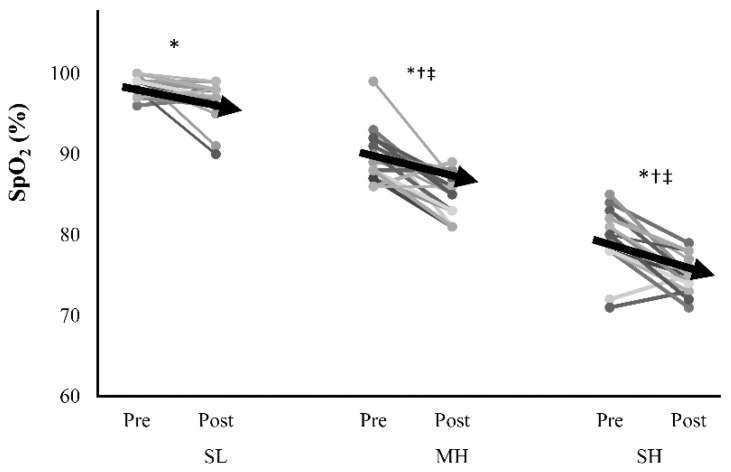
Peripheral oxygen saturation (SpO_2_) responses to sprint interval training exercise. Arrows are means, and lines present individual responses. SL: sea level; MH: moderate hypoxia; SH: severe hypoxia; PP: peak power; MP: mean power. * Statistically different from the corresponding Pre values (*p* < 0.05). † Statistically different from the corresponding SL values (*p* < 0.05). ‡ Statistically different from the corresponding MH values (*p* < 0.05).

There were statistically differences in HR and SpO_2_ (i.e., normobaric normoxia, moderate hypoxia, and severe hypoxia) within (i.e., pre to post) and between conditions (Table 1 and Figure 1). Before exercise, MH mean HR values were statistically higher than SL mean values, and SH mean values were statistically higher than both MH and SL mean values. However, during exercise no statistically condition differences were found for mean HR (SL 84 ± 7% vs. MH 85 ± 6% vs. SH 85 ± 7%, *p* = 0.928, *ƞ_p_*^2^ = 0.009) and the highest HR (SL 93 ± 4% vs. MH 93 ± 6% vs. SH 93 ± 5%, *p* = 0.957, *ƞ_p_*^2^ = 0.005) For SpO_2_, all conditions produced statistically detectable pre to post decreases. Before exercise, MH mean SpO_2_ values were statistically lower than SL mean values, and SH mean values were statistically lower than both MH and SL mean values (Figure 1). 

**Figure 2:**
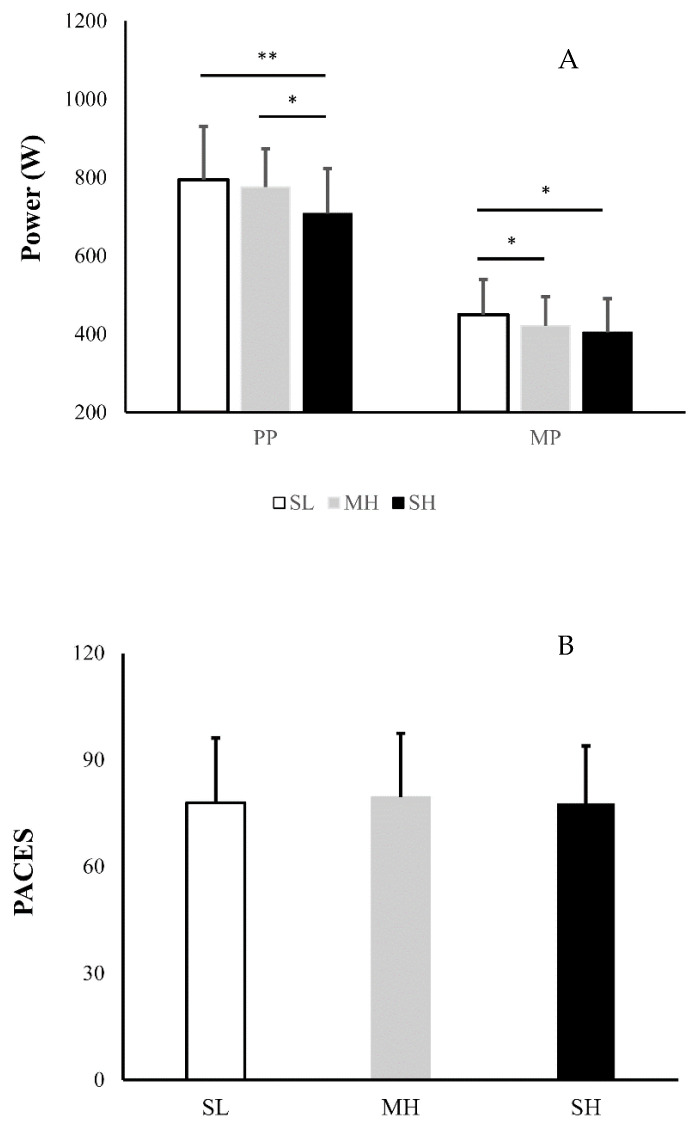
Power outputs during exercise (**A**) and scores of physical activity enjoyment scale (PACES) (mean and standard deviation) (**B**) after sprint interval exercise at different hypoxia. * *p* **<** 0.05, **p < 0.01. SL: sea level; MH: moderate hypoxia; SH: severe hypoxia; PP: peak power; MP: mean power.

Peak power output was statistically lower in the SH compared to SL (*p* = 0.018, *d* = 0.72) and MH (*p* = 0.046, *d* = 0.67), whereas no statistical difference between SL and MH was found between SL and SH (Figure 2A). Mean power output was statistically higher in SL compared to MH (*p* = 0.027, *d* = 0.34) and SH (*p* = 0.019, *d* = 0.51), while SH and MH were statistically similar (Figure 2B). 

For RPE, all conditions produced statistically detectable pre to post increases with no between condition statistical differences and no interaction effects.

### 3.2. Affective Responses

As shown in Table 1, there were no pre to post statistical differences in affect valence across conditions as indicated by FS scores, but statistically higher pre to post activation were observed in SL (4.0 ± 1.2), MH (3.9 ± 1.4), and SH (3.6 ± 1.4) (*p* <0.05). According to the circumplex model [22], participants were generally calm before exercise (low activation, positive valence) and reported a greater sense of energy and vigor after exercise (higher activation, positive valence).

For enjoyment responses indicated by EES (Table 1), no statistical differences were found from pre to post exercise. EES scores immediately end SIT indicated a medium level of enjoyment (SL: 3.9 ± 1.5; MH: 3.8 ± 1.3; SH: 3.8 ± 1.8 based on a high score of 7). PACES scores indicated a medium level of enjoyment after SIT under the three conditions (SL: 78 ± 18; MH: 80 ± 18; SH: 78 ± 18) and there were also no statistical differences in PACES scores (*p* = 0.759) across the three conditions (Figure 2B).”

## 4. Discussion

Although affective responses to SIT under normoxia have been analyzed in the current literature, the present study firstly addressed the issue regarding affective responses to SIT under hypoxic conditions. Contrary to our hypothesis that more negative affective responses to SIT would be triggered under severe hypoxic condition, our results showed that there were no statistical differences in affective responses to SIT under normoxia, moderate hypoxia, and severe hypoxia, suggesting that affective responses to SIT were not impacted by oxygen extraction under hypoxia.

Comparable relative intensity across the three conditions might help to explain the similar affective responses. Although SpO_2_ (%) and power output were affected by hypoxic conditions, HR during exercise showed no statistical differences between trials, indicating that all trials produced similar physiological stress. Therefore, similar affective responses across the conditions were not unexpected when same SIT protocol were performed with nearly the same relative intensity. Similar perceived exertion may also account for the lack of differences in affective responses across trials. Inconsistent with the current findings, several studies reported that performing SIT under severe hypoxia was associated with elevated RPE and exacerbated exercise-related sensations such as subjective fatigue and discomfort [16,18,31]. Although exercise-related sensations were not measured in the current study, our results did not show a higher RPE in the SH compared to the other two oxygen conditions. Yet, it should be noted that RPE scores were relatively high in all trials, indicating a ‘very severe’ (RPE = 7/10) level of difficult breathing.

The circumplex model which combines affect valence and felt of arousal could provide a comprehensive analysis of affective responses [22,32]. From the perspective of the circumplex model, severe hypoxic SIT as well as SIT under moderate hypoxia and normoxia generated a sense of energy (positive affect and high arousal) immediately after exercise. It is interesting that affect valences immediately after exercise were positive (i.e., above 0) regardless of the oxygen environment in the current study. Based on data of previous studies carried out under normoxic condition, affect valences tended to reach the lowest point immediately post exercise [14,33,34,35]. Some studies reported negative affect valences at the very end of SIT sessions [14,33], while results in other studies showed that affect valences remained positive [34,35]. Exercise intensity is a primary factor influencing affective responses in continuous training according to the dual-mode theory [22], yet affective responses to SIT with all-out effort could be influenced by other factors, for example, varied methodologies applied across studies. Work-to-rest ratio and sprint duration might be essential variables that caused differences in perceptions to SIT, as negative affect valences have been reported in studies of SIT with shorter recovery but longer work duration [36,37]. However, based on existing data with contradictory results in the literature [14,33,38], there is still no solid evidence to draw conclusions. Individual differences in physical (e.g., physical activity level [39]), psychological status (e.g., exercise preference and tolerance of intensity) [40,41], and macronutrient intake (e.g., low-carbohydrate diets) [42] also account for the discrepancy. From the aspect of the neural system, the interplay of prefrontal cortex and amygdala have been revealed to regulate affective responses during exercise [43,44,45]. For example, individual differences in the ability to maintain prefrontal cortex involvement, indicated by self-reported tolerance, are possibly associated with feelings of pleasure or displeasure [41,44]. Nevertheless, examinations of individual’s cognitive and neural characteristics and how these characteristics influence affective responses to SIT were not included in the current study, which might be a future study focus to better interpret the results.

In the present study, post exercise enjoyment responses were measured through EES and PACES, and both scales showed no statistical differences across the three conditions. However, inconsistent with the present results, enjoyment responses were more positive (i.e., ~100 points) in a previous study incorporating a similar SIT protocol (i.e., 5 s of sprinting and 40 s of passive rest for a maximum of 24 repetitions) under normoxia in active males [14]. Measurement time could be critical to explicate the uneven enjoyment level as scales of enjoyment were taken immediately post exercise in the present study but 30-min post exercise in the aforementioned study. There might be a rebound effect in enjoyment level after participants remedied from the intense SIT with adequate rest. Importantly, affective responses immediately after exercise and the lowest point of affective responses, which often concurrently existed, could provide more meaningful clues to predict future behavior or exercise adherence, as profound memories of exercise experiences were formed in these moments [46].

Contrary to our findings, a recent study [31] of trained athletes reported statistical lower level of pleasure after a high-intensity interval running protocol with four sets of 4-min intervals at a RPE of 16 on 6–20 Borg scale in a moderate hypoxic condition (FiO_2_ = 15.0%) compared with normoxia (FiO_2_ = 20.9%). It is possible that performing high-intensity exercise with a considerable long work duration and total exercise duration under hypoxia might intensify cerebral deoxygenation [47,48] and trigger unpleasant feelings. Specifically, the HIIT protocol with 4-min could place heavier reliance on oxidative metabolism than SIT with less than 10-s intervals which rely more on anaerobic system but peripheral aerobic system, thus oxygen deficits under hypoxia could be more severe in long-duration HIIT in comparison to SIT [49]. Therefore, despite exercising hypoxia possibly leading to additional metabolic adaptions and health improvements, it could be crucial to explore which configuration of HIIT is well-tolerated in oxygen restricted environment to avoid possible adverse exercise perceptions and negative impacts on future adherence. Given that favorable and comparable affective responses to a session of SIT under severe hypoxia in comparison to normoxia and moderate hypoxia were observed in the current study, it is fair to speculate that adding a hypoxic condition to the adopted SIT protocol might induce a similar level of future adherence as normoxia SIT based on the hedonic theory of behavior [8]. Nevertheless, long-term investigations on SIT with practical designs in a wider range of population groups are warranted to further confirm the applications of the SIT under severe hypoxia.

The current study took the first attempt to analyze affective responses under different hypoxic conditions. Our study was strengthened by using randomized within-subject design to make direct comparisons in affective responses to SIT under different hypoxia conditions. Moreover, daily physical activity and diet were well-controlled during the experiment by using validated pedometers and providing standard meals to the participants. However, several limitations should be mentioned to interpret the results. Firstly, participants recruited in the current study were recreational active males, which limits the applications to other populations, especially for those with limited exercise experiences and low fitness level. Secondly, fluctuation of affect, peak negative affect during exercise could provide meaningful interpretations to the affective responses, yet in-task affective responses were not measured in the present study. Thirdly, as the current data only demonstrated acute affective responses to SIT in hypoxia, future studies could benefit from applying a long-term intervention. Fourthly, the basic psychological characteristics prior to the tests (e.g., state and anxiety traits or personality traits) might affect the affective and enjoyment responses to exercise. This interesting question awaits furthers investigation. Lastly, cautions should be mentioned when form generalizations based on the current results, as there were considerable variations in SIT protocol designs and differences in experimental subjects.

## 5. Conclusions

Given that direct comparisons were made across the conditions as participants performed SIT exercise under all three oxygen conditions in a randomized order, it seems reasonable to conclude that practicing SIT under severe hypoxia could be as pleasurable and enjoyable as the practices at sea level or less intense hypoxic condition in active individuals.

## Figures and Tables

**Table 1:** Physiological and psychological responses to sprint interval exercise at different hypoxia.

SL					MH				SH			
	Pre		Post		Pre		Post		Pre		Post	
HR (bpm)	68	±8	150	±12 *	72	±8 ^†^	155	±12 *	79	±11 ^†^^‡^	152	±13 *
SpO_2_ (%)	98	±1	96	±1 *	90	±3 ^†,^^‡^	86	±3 *	79	±3 ^†^^‡^	75	±3 *
RPE	0.4	±0.8	7.2	±2.9 *	0.3	±0.4	6.8	±2.2 *	0.6	±1.0	7.4	±2.4 *
FS	1.7	±1.3	1.7	±1.5	1.0	±1.7	1.1	±1.5	1.2	±1.3	1.4	±1.3
FAS	2.3	±1.2	4.0	±1.2 *	2.6	±1.5	3.9	±1.4 *	2.6	±1.4	3.6	±1.4 *
EES	4.0	±2.0	3.9	±1.5	3.7	±1.9	3.8	±1.3	4.2	±0.7	3.8	±1.8

SL: sea level; MH: moderate hypoxia; SH: severe hypoxia. RPE: ratings perceived exertion; FS: feeing scale; FAS: felt arousal scale; EES: exercise enjoyment scale. * Statistically different from the corresponding Pre values (*p* < 0.05). ^†^ different from the corresponding SL values (*p* < 0.05). ^‡^ Statistically different from the corresponding MH values (*p* < 0.05).

## Data Availability

The data presented in this study are available on request from the corresponding author. The data are not publicly available due to the wishes of some subjects.
